# Nausea and vomiting of pregnancy: associations with personal history of nausea and affected relatives

**DOI:** 10.1007/s00404-020-05683-3

**Published:** 2020-07-11

**Authors:** Linda Laitinen, Miina Nurmi, Pauliina Ellilä, Päivi Rautava, Mari Koivisto, Päivi Polo-Kantola

**Affiliations:** 1 Department of Obstetrics and Gynecology, Central Finland Central Hospital, Keskussairaalantie 19, 40620 Jyväskylä, Finland; 2 University of Turku, Turku, Finland; 3 Department of Obstetrics and Gynecology, Turku University Hospital and University of Turku, Turku, Finland; 4 Department of Public Health, University of Turku, Turku, Finland; 5 Turku Clinical Research Centre, Turku University Hospital, Turku, Finland; 6 Department of Biostatistics, University of Turku, Turku, Finland

**Keywords:** Nausea and vomiting of pregnancy, PUQE, Pregnancy, Migraine, Relatives

## Abstract

**Purpose:**

To examine whether personal history of nausea or history of nausea and vomiting of pregnancy (NVP) in relatives are risk factors for a woman to suffer from NVP. Further, to evaluate if these factors are associated with the severity of NVP.

**Methods:**

Cohort study of 2411 pregnant women recruited from maternity health care clinics. The severity of NVP was categorized according to Pregnancy-Unique Quantification of Emesis (PUQE) questionnaire into no/mild/moderate/severe NVP. History of nausea was assessed in connection with motion sickness, seasickness, migraine or other kinds of headache, after anesthesia, related to the use of contraceptives, and other kinds of nausea. History of NVP in relatives was categorized into first-degree (mother/sister) and second-degree relatives (more distant).

**Results:**

In multivariable analysis including previous personal history of nausea, motion sickness (OR 3.17, 95% CI 1.81–5.56, *p* < 0.0001) and nausea in migraine (OR 3.18, 95% CI 1.86–5.45, *p* < 0.0001) were associated with severe NVP. History of nausea in other kinds of headache was associated with moderate NVP (OR 1.91, 95% CI 1.34–2.72, *p* = 0.001). Women with affected first-degree relatives had higher odds for moderate (OR 3.84, 95% CI 2.72–5.40) and severe (OR 3.19, 95% CI 1.92–5.28) NVP (*p* < 0.0001). All these results remained significant after adjusting for parity, body mass index, smoking, employment and age.

**Conclusion:**

Women with personal history of nausea or family history of NVP have an increased susceptibility of NVP. This information is useful in pre-pregnancy counselling.

**Electronic supplementary material:**

The online version of this article (10.1007/s00404-020-05683-3) contains supplementary material, which is available to authorized users.

## Introduction

Nausea and vomiting of pregnancy (NVP) affect up to 80% of women in early pregnancy [1]. Symptoms vary from mild nausea to severe form of NVP, which is called hyperemesis gravidarum (HG) with excessive vomiting, dehydration, electrolyte imbalances, and weight loss [2]. NVP decreases the quality of life and has major economic influence causing often absence from work [3]. In addition, HG is the second most common reason for hospitalization during pregnancy after preterm birth [2].

Etiology of nausea in general [4] seems to be multifactorial, and the same applies for the etiology of NVP [5, 6]. Many women experience nausea and vomiting in connection with motion sickness, seasickness, migraine or other kind of headache [7–10]. Nausea and vomiting are also reported in relation to the use of oral contraceptives and after anesthesia [11, 12]. Mechanisms behind both general nausea and NVP involve vestibular, olfactory, hormonal and gastrointestinal stimuli which are processed in central nervous system [4–6, 9]. Accordingly, women with history of nausea in connection with various events or diseases may be prone to NVP. However, this issue is not thoroughly covered in previous literature. In addition, several studies have suggested genetic predisposition to NVP and HG [13–16] and new data of genetics is emerging [17].

The aim of our study was to evaluate whether previous history of nausea or history of NVP in relatives were associated with an increased occurrence of NVP. Further, we hypothesized that these factors are associated with the severity of NVP. The severity of NVP symptoms can be evaluated with a simple and validated tool, pregnancy-unique quantification of emesis questionnaire (PUQE) [18], which we thus used in our cohort.

## Material and methods

Women were enrolled from 33 maternity health care clinics (MHCCs) from Turku city area and surrounding municipalities. More than 99% of women use the public MHCC services during pregnancy in Finland [19]. MHCC nurses, carefully instructed by researchers, recruited the women during their routine mid-pregnancy visits between October 2011 and November 2014. Annually during the study period, the amount of the deliveries varied from 4698 to 4812 in Hospital District of Southwest Finland [19]. After receiving oral and written information about the study, women filled in the study questionnaire. This was also considered as informed consent. The study had approval from the Joint Ethics Committees of University of Turku and Turku University Central Hospital, Turku, Finland (43/180/2011).

**Fig.1:**
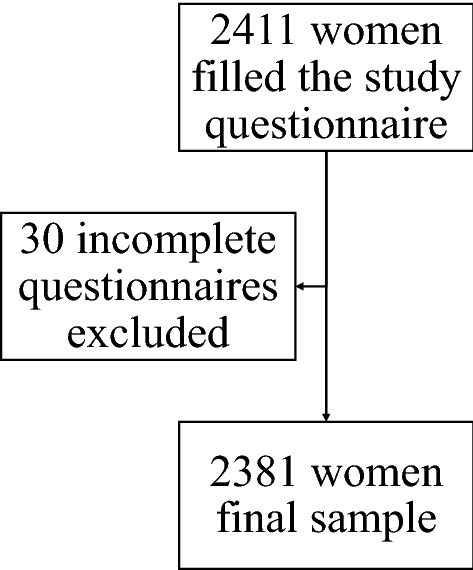
Flowchart of the study

Study population consisted of 2411 women. Questionnaire was incomplete in 30 women leaving 2381 women into analysis (mean age 30.3, SD 4.7, range 15.2–45.9) (Fig. 1). NVP was evaluated with Motherisk PUQE scoring system [18]. PUQE questionnaire was translated into Finnish with permission of the PUQE owners by professional translator and retranslated into English by another professional translator. PUQE questionnaire consists of three short questions; daily duration of nausea in hours (question 1), number of vomits (question 2) in 12 h and number of retching (question 3) in 12 h. Answers are rated from scale of 1–5, where higher points signify more severe symptoms. In the question 2 answers were reversed because item is phrased in descending order. PUQE total score is the sum of the replies, according to which the severity of NVP is rated as ‘no NVP’ (3 points), ‘mild’ (4–6 points), ‘moderate’ (7–12 points) and ‘severe’ (13–15 points)^16^. Mean gestational week (gwk) at the response was 20.2 (range 7–40). Women were asked to reply according to the worst 12 h of NVP of their pregnancy. Because in most of the women the worst NVP had already relieved when answering the study questionnaire, to avoid recalling bias, we conducted a sub-analysis including only women who had replied ≤ 20 gwk.

Personal history of nausea in various situations was asked (yes/no) concerning motion sickness, seasickness, migraine, other kind of headache, after anesthesia, during the use of contraception and other kind of nausea. Method of contraception and context of other kind of nausea were specified with an open question. NVP of relatives was asked (yes/no/not known and who) and the answers of affected relatives were categorized into two groups: the first-degree relatives (mother, sister) and the second-degree relatives (grandmother, aunt, cousins, more distant relatives).

Medical Birth Register of Finnish Institute for Health and Welfare was used to collect basic characteristics of the women: parity (nullipara/multipara), pre-pregnancy body mass index (BMI, kg/m^2^) calculated by weight and height, smoking (no/yes), marital status (cohabited/single) and employment (working/not working). Age (years) was calculated by comparing the date of birth of the mother to the answering date (Table 1).

**Table 1:** Basic characteristics (total *n* = 2381)

	*n*	Mean ± SD	Range
		or % (*n*)	
Age (years)	2363	30.3 ± 4.7	15.2–45.9
Parity	2325		
Nulliparous		46.0 (1069)	
Multiparous		54.0 (1256)	
BMI (kg/m^2^)	2324	24.6 ± 4.8	15.1–57.8
Smoking	2319		
Non-smokers		87.0 (2018)	
Smokers		13.0 (301)	
Marital status	2306		
Cohabited		96.2 (2218)	
Single		3.8 (88)	
Employment	2043		
Working		83.1 (1697)	
Not working		16.9 (346)	

### Statistical analysis

Continuous variables were characterized using means, standard deviations (SD) and ranges of values. In case of categorical variables, frequencies and percent were used. NVP (total score of three PUQE questions) was categorized into four categories according to the severity of NVP (‘no NVP’, ‘mild’, ‘moderate’ and ‘severe’). First, univariate analysis (cumulative logit regression analysis) between categorized NVP and various variables of personal history of nausea and family history of NVP was conducted separately. Secondly, the results were adjusted for parity, BMI, smoking, employment and age. Thereafter, multivariable analysis (cumulative logit regression analysis) was carried out to study the associations between categorized NVP and all variables of personal history of nausea. The same analyses were performed to study associations between each PUQE question and variables of personal history of nausea and family history of NVP. In addition, subgroup analyses were repeated including only women answering in $\le$ 20 gwk. The results are presented with *p* values, OR and 95% CI. Statistical analyses were carried out using a 9.4 version of SAS Institute Inc. (Cary, NC, USA) for Windows, and *p *values of < 0.05 were considered as statistically significant.

## Results

Altogether 88% of 2381 women suffered from NVP. Of all women, 29.4% (*n* = 700) had mild, 52.2% (*n* = 1243) had moderate and 6.4% (*n* = 152) had severe NVP, and 12.0% (*n* = 286) women stated having no NVP. Majority of women (65.4%) reported daily duration of nausea ≥ 2 h, and in 45.0% the daily duration was ≥ 4 h. Further, 18.7% of women had vomiting ≥ 3 times in 12 h, whereas 37.6% of women had retching ≥ 3 times in 12 h.

From the entire study cohort, 47.0% (*n* = 1091) of women suffered from motion sickness, 32.1% (*n* = 731) had seasickness, 30.2% (*n* = 687) had migraine, 40.1% (*n* = 904) had history of other kind of headache and 12.5% (*n* = 262) had history of nausea after anesthesia. Nausea during the use of contraception was reported by 4.4% (*n* = 95) women. Method of contraception included combined oral contraceptives (*n* = 61), contraceptive pills (non-specified, *n* = 13), levonorgestrel-releasing intrauterine device (*n* = 4), progestin-only pills (*n* = 4), contraceptive patch (*n* = 4), contraceptive vaginal ring (*n* = 3) and emergency contraception (*n* = 2). 15.4% (*n* = 271) of women specified the context of other kind of nausea; in rotating motion for example in amusement park (*n* = 27), during gastroenteritis or other illness (*n* = 26), hunger (*n* = 24), pain (*n* = 20) or with repulsive odors (*n* = 18).

In univariate analysis, history of motion sickness, seasickness, migraine and other kind of headache were associated with more severe NVP. Further, history of nausea during the use of contraception, after anesthesia and other kind of nausea were associated with moderate and severe NVP. After adjusting by parity, BMI, smoking, employment and age, the results remained the same. In multivariable analysis, severity of NVP was associated with the history of motion sickness. Instead, history of migraine was associated only with severe NVP and history of other type of headache only with moderate NVP (Table 2).

**Table 2:** Associations between PUQE total score and history of nausea

	*n*	*p*^*a*^	*p*^*b*^	*p*^*c*^	No NVP	Mild NVP						Moderate NVP						Severe NVP					
						Univariate				Multivariable		Univariate				Multivariable		Univariate				Multivariable	
					OR	OR	95% CI	AOR^b^	95% CI	OR	95% CI	OR	95% CI	AOR^b^	95% CI	OR	95% CI	OR	95% CI	AOR^b^	95% CI	OR	95% CI
Motion sickness	1091	< *0.001*	< *0.0001*	< *0.0001*	1	1.82	1.35–2.46	1.67	1.21–2.31	1.59	1.06–2.40	2.65	2.0–3.52	2.54	1.87–3.45	2.19	1.49–3.23	3.81	2.50–5.81	3.93	2.48–6.23	3.17	1.81–5.56
Seasickness	731	*0.0003*	*0.0003*	*0.20*	1	1.81	1.29–2.53	1.72	1.20–2.47	1.29	0.82–2.02	1.96	1.43–2.69	1.94	1.37–2.74	1.02	0.67–1.57	2.29	1.47–3.57	2.65	1.63–4.31	0.78	0.42–1.46
Migraine	687	< *0.0001*	< *0.0001*	< *0.0001*	1	1.19	0.85–1.66	1.28	0.89–1.84	1.03	0.67–1.58	1.76	1.29–2.41	1.79	1.27–2.51	1.45	0.98–2.16	3.39	2.19–5.24	4.23	2.62–6.84	3.18	1.86–5.45
Other headacne	904	< *0.0001*	< *0.0001*	*0.001*	1	1.70	1.24–2.34	1.66	1.17–2.35	1.42	0.98–2.07	2.46	1.83–3.32	2.50	1.80–3.47	1.91	1.34–2.72	2.30	1.50–3.54	2.32	1.44–3.73	1.42	0.84–2.39
After anesthesia	262	*0.014*	*0.04*	*0.69*	1	1.27	0.77–2.10	1.19	0.70–2.04	0.98	0.56–1.73	1.79	1.12–2.86	1.64	1.00–2.71	1.20	0.71–2.03	2.10	1.11–3.99	2.09	1.05–4.17	1.26	0.59–2.68
Contraception	95	*0.003*	*0.007*	*0.27*	1	2.80	0.83–9.45	2.22	0.64–7.65	3.98	0.51–31.17	4.83	1.50–15.52	3.72	1.14–12.10	5.94	0.80–44.17	7.57	2.07–27.62	6.87	1.84–25.59	5.55	0.6–51.39
Other kind of nausea	271	*0.033*	*0.045*	*0.16*	1	1.47	0.89–2.43	1.33	0.77–2.28	1.51	0.86–2.66	1.83	1.14–2.94	1.73	1.04–2.87	1.73	1.01–2.97	2.23	1.17–4.23	2.30	1.15–4.58	2.07	1.01–4.25

*AOR* adjusted odds ratio: adjusted for parity, *BMI* body mass index, smoking, employment, age, *NVP* Nausea and vomiting of pregnancy, *PUQE* Pregnancy-Unique Quantification of Emesis Questionnaire

^a^Univariate analysis (cumulative logit regression analysis)

^b^Adjusted for parity, body mass index (BMI), smoking employment, age

^c^Multivariable analysis including all history of nausea variables (cumulative logit regression analysis)

In adjusted analysis of each PUQE question at a time (daily duration of nausea, number of vomits and number of retching), history of motion sickness, seasickness and migraine were associated with all aspects of NVP. In addition, history of other kind of headache was associated with longer daily duration of nausea and higher number of retching. Women with history of nausea after anesthesia had higher odds for daily duration of nausea over 6 h and those having other kind of nausea higher odds for daily duration of nausea over 4 h. Further, women who had suffered nausea during contraception use had higher odds for duration of nausea over 4 h and for over 7 vomiting episodes (Table 3).

**Table 3:** Adjusted associations between history of nausea and different PUQE –questions

	Motion sickness *p*		Seasickness *p*		Migraine *p*		Other headache *p*		After anesthesia *p*		Contraception *p*		Other kind of nausea *p*	
	AOR	95% CI	AOR	95% CI	AOR	95% CI	AOR	95% CI	AOR	95% CI	AOR	95% CI	AOR	95% CI
Duration of nausea (Question 1)	< *0.0001*^*a*^		*0.0004*^*a*^		< *0.0001*^*a*^		< *0.0001*^*a*^		*0.005*^*a*^		*0.029*^*a*^		*0.017*^*a*^	
None	1		1		1		1		1		1		1	
1 h or less	1.41	1.02–1.96	1.52	1.06–2.20	1.56	1.08–2.27	1.59	1.13–2.24	1.20	0.69–2.09	2.79	0.79–9.92	1.31	0.76–2.26
2–3 h	1.98	1.43–2.73	1.73	1.20–2.49	1.61	1.11–2.33	1.71	1.21–2.42	1.26	0.73–2.19	3.06	0.87–10.81	1.23	0.71–2.12
4–6 h	2.98	2.08–4.26	2.01	1.35–2.98	1.62	1.08–2.43	2.59	1.79–3.77	1.45	0.80–2.61	4.61	1.29–16.46	1.90	1.07–3.39
Over 6 h	2.65	1.95–3.60	2.09	1.49–2.93	2.39	1.70–3.38	2.38	1.72–3.28	2.11	1.29–3.46	5.26	1.59–17.38	1.97	1.20–3.24
Vomiting (Question 2)	*0.0001*^*a*^		*0.008*^*a*^		< *0.0001*^*a*^		*0.077*^*a*^		*0.057*		*0.039*^*a*^		*0.55*	
Not once	1		1		1		1		1		1		1	
1–2 times	1.47	1.19–1.82	1.17	0.93–1.47	1.12	0.88–1.41	0.93	0.74–1.16	0.91	0.64–1.30	1.37	0.79–2.37	1.14	0.82–1.59
3–4 times	1.36	1.00–1.85	1.07	0.76–1.52	1.59	1.15–2.21	1.48	1.08–2.03	1.41	0.89–2.25	1.49	0.69–3.19	1.18	0.72–1.95
5–6 times	1.70	1.07–2.70	2.02	1.26–3.23	1.85	1.16–2.96	0.99	0.61–1.58	1.15	0.55–2.38	1.70	0.58–4.95	1.22	0.60–2.47
7 times or more often	2.02	1.32–3.12	1.74	1.12–2.71	3.40	2.20–5.28	1.27	0.82–1.95	2.09	1.18–3.72	3.45	1.58–7.51	1.68	0.90–3.14
Retching (Question 3)	< *0.0001*^*a*^		*0.024*		< *0.0001*^*a*^		< *0.0001*^*a*^		*0.099*^*a*^		*0.21*		*0.11*	
Not once	1		1		1		1		1		1		1	
1–2 times	1.31	1.04–1.65	1.11	0.86–1.42	1.19	0.92–1.53	1.51	1.19–1.92	1.30	0.90–1.88	1.48	0.80–2.76	1.13	0.78–1.64
3–4 times	1.58	1.21–2.07	1.39	1.04–1.86	1.28	0.95–1.72	1.61	1.22–2.13	1.31	0.85–2.02	1.34	0.65–2.78	1.43	0.94–2.19
5–6 times	1.96	1.36–2.82	1.63	1.10–2.42	1.56	1.06–2.30	1.74	1.19–2.54	1.85	1.07–3.19	2.21	0.98–5.01	1.89	1.11–3.20
7 times or more often	1.95	1.47–2.59	1.42	1.05–1.91	2.48	1.85–3.33	1.85	1.39–2.47	1.63	1.06–2.51	2.03	1.02–4.02	1.41	0.90–2.19

*AOR*  Adjusted odds ratio: adjusted for parity, body mass index (BMI), smoking, employment, age, *PUQE* Pregnancy-Unique Quantification of Emesis Questionnaire

^a^Significant in univariate analysis

In the subgroup analysis of women answering ≤ 20 gwk (*n* = 1247), the percentages of women in different PUQE categories were practically the same as in the entire study cohort (data not shown). In the univariate analysis, history of motion sickness, seasickness, migraine and other kind of headache were associated with the severity of NVP. After adjusting by parity, BMI, smoking, employment and age, the results remained the same. In multivariable analysis, severity of NVP was associated with history of motion sickness, migraine and other kind of headache. (Online resource 1). In adjusted analysis of each PUQE question at a time (daily duration of nausea, number of vomits and number of retching), history of motion sickness and migraine were associated with all aspects of NVP. In addition, history of seasickness was associated with longer duration of nausea and ≥ 5 vomiting episodes. Further, history of other headache was associated with duration of nausea ≥ 4 h and ≥ 7 episodes of retching. History of nausea after anesthesia was associated with ≥ 7 vomiting episodes. (Online resource 2).

Of all women, 874 (37.5%) women had affected first-degree relatives and 60 (2.6%) women had affected second-degree relatives. Of all women, 481 (20.7%) stated not having an affected relative and 913 (39.2%) reported not knowing family history of NVP. History of NVP in affected first-degree relatives was associated with more severe NVP. However, history of NVP in affected second-degree relatives was associated with moderate and severe NVP. When analyzing each PUQE question at a time (daily duration of nausea, number of vomits and number of retching), all aspects of NVP except vomiting ≥ 7 times in 12 h were associated with history of NVP in affected first-degree relatives. Women who had affected second-degree relatives with history of NVP had higher odds for daily duration of nausea ≥ 4 h and vomiting ≥ 7 times in 12 h and they also had higher odds for retching ≤ 2 times in 12 h (Table 4). The results remained significant also when adjusting by parity, BMI, smoking, employment and age.

**Table 4:** History of NVP in relatives, PUQE total score and different PUQE–questions

	*p*^*a*^	*p*^*b*^	No	First-degree relatives				Second–degree relatives				Not known			
			*n* = 481	*n* = 874				*n* = 60				*n* = 913			
			OR	OR	95% Cl	AOR	95% CI	OR	95% Cl	AOR	95% CI	OR	95% CI	AOR	95% CI
PUQE total score	< *0.0001*	< *0.0001*													
No			1	1		1		1		1		1		1	
Mild				2.06	1.43–2.97	2.04	1.37–3.03	1.54	0.52–4.55	1.37	0.45–4.16	2.43	1.73–3.42	2.44	1.68–3.52
Moderate				3.84	2.72–5.40	4.23	2.91–6.15	3.97	1.51–10.40	3.16	1.17–8.53	2.78	2.00–3.85	2.99	2.09–4.27
Severe				3.19	1.92–5.28	3.56	2.05–6.20	4.28	1.28–14.38	3.84	1.08–13.62	1.14	0.66–1.99	1.26	0.68–2.31
Duration of nausea (Question 1)	< *0.0001*	< *0.0001*													
None			1	1		1		1		1		1		1	
1 h or less				1.94	1.34–2.81	1.72	1.15–2.56	1.36	0.46–4.04	1.17	0.35–3.97	1.91	1.34–2.70	1.80	1.23–2.63
2–3 h				2.21	1.52–3.23	2.21	1.47–3.32	1.59	0.53–4.73	1.83	0.57–5.83	2.31	1.62–3.31	2.31	1.57–3.41
4–6 h				5.06	3.16–8.10	5.97	3.56–10.01	6.65	2.32–19.04	5.78	1.74–19.19	4.21	2.66–6.65	4.62	2.77–7.69
Over 6 h				3.42	2.42–4.84	3.68	2.51–5.37	3.83	1.52–9.63	4.19	1.51–11.58	2.03	1.44–2.84	2.21	1.52–3.21
Vomiting (Question 2)	< *0.0001*	< *0.0001*													
Not once			1	1		1		1		1		1		1	
1–2 times				2.06	1.56–2.72	2.06	1.52–2.78	1.55	0.79–3.06	1.68	0.80–3.52	1.49	1.13–1.97	1.46	1.08–1.98
3–4 times				1.57	1.06–2.31	1.62	1.07–2.46	1.41	0.55–3.58	1.71	0.65–4.49	0.99	0.67–1.47	1.02	0.67–1.57
5–6 times				1.81	1.03–3.20	1.90	1.02–3.53	2.29	0.73–7.23	2.19	0.59–8.17	0.71	0.38–1.34	0.84	0.43–1.67
7 times or more often				1.56	0.96–2.52	1.48	0.88–2.49	2.68	1.07–6.68	2.24	0.78–6.46	0.56	0.32–0.97	0.56	0.31–1.02
Retching (Question 3)	< *0.0001*	*0.0001*													
Not once			1	1		1		1		1		1		1	
1–2 times				2.01	1.50–2.69	2.04	1.48–2.81	2.44	1.22–4.90	2.45	1.13–5.32	1.64	1.23–2.18	1.70	1.25–2.32
3–4 times				2.24	1.59–3.16	2.36	1.62–3.43	2.33	1.04–5.23	2.75	1.15–6.58	1.40	0.99–1.98	1.58	1.08–2.29
5–6 times				2.24	1.41–3.57	2.44	1.46–4.06	2.26	0.78–6.59	2.35	0.71–7.76	1.57	0.99–2.51	1.59	0.95–2.66
7 times or more often				1.95	1.38–2.75	1.98	1.37–2.86	1.61	0.67–3.91	1.49	0.55–4.06	1.12	0.79–1.59	1.17	0.80–1.70

*AOR* Adjusted odds ratio: adjusted for parity, *BMI* body mass index, smoking, employment age, *NVP* nausea and vomiting of pregnancy, *PUQE* pregnancy-unique quantification of emesis questionnaire

^a^Univariate analysis

^b^Adjusted for parity, body mass index (BMI), smoking, employment, age

In the subgroup analysis, history of nausea in affected first-degree relatives was associated with more severe NVP, and history of NVP in affected second-degree relatives was associated with moderate and severe NVP. In adjusted analysis of each PUQE question at a time, there was an association between NVP of affected first-degree relatives and daily duration of nausea ≥ 2 h. In women with affected first-degree relatives, there were increased odds for vomiting ≤ 2 times compared to women with no vomiting but in women vomiting ≥ 3 times the association was not more statistically significant. Women with affected second-degree relatives had increased odds for duration of nausea ≥ 4 h. Concerning retching, there were increased odds for more retching episodes in women with affected first-degree relatives but not in women with affected second-degree relatives. (Online resource 2).

## Discussion

To best of our knowledge, our study is the first to address the connection with personal history of nausea in various situations and the severity of NVP assessed by PUQE. We confirmed our hypothesis that several aspects of personal history of nausea were linked with the severity of NVP, particularly the history of motion sickness, migraine and other type of headache. The same held true with different characteristics of NVP (daily duration of nausea, number of vomiting and retching). This finding suggests that the factors behind NVP stem from similar factors as nausea and vomitus in general. Therefore, assessing the history of nausea and vomitus may be used in pre-pregnancy counselling assessing susceptibility to NVP. In addition, history of NVP in relatives of the pregnant woman was associated with increased odds of having NVP. This supports the hypothesis of the genetic predisposition to the condition.

Nausea and vomiting are triggered in the vomiting center in central nervous system, an area in brainstem which is sensitive to various stimuli [4, 6]. Although the different types of nausea which we assessed in our study manifest also as distinct conditions, they probably share common or interacting mechanisms. For instance, female gender, history of motion sickness and migraine are known risk factors for nausea after anesthesia [20]. Furthermore, women are more susceptible to motion sickness and to its variant seasickness [7], as well as to migraine and other headache [10] than men. Indeed, female sex hormones, estrogen and progesterone, have been linked to migraine [21] and to nausea during the use of hormonal contraception [11, 22]. During pregnancy, serum estrogen and progesterone levels increase rapidly early in the first trimester, and thus, may contribute to NVP and HG [23].

Motion sickness and its variant seasickness are thought to arise from disturbances between visual stimuli, proprioception and the vestibular system [7, 8]. Connections between motion sickness and NVP have been described already three decades ago [24–26] although recent literature has not carried out the issue. Järnefelt-Samsioe et al. [24] found no difference in the frequency of motion sickness between emetic and non-emetic women but symptoms of motion sickness were aggravated during pregnancy in emetic women. However, Whitehead et al. and Gadsby et al. [25, 26] found higher reported frequency of motion sickness in women with NVP. Goodwin et al. [27] found in their preliminary study using vestibular autorotation test abnormalities in vestibular system in patients with HG. Some HG patients demonstrated subclinical abnormalities in vestibuloocular reflex also postpartum [27]. This could support the hypothesis of unmasking of subclinical vestibular disorders behind some cases of HG [27, 28]. We found association between NVP and both history of motion sickness and seasickness. The association between seasickness and NVP was not significant in multivariable analysis probably because of its close relation to motion sickness.

In women, migraine is not only more common and severe but also more often connected with associated symptoms, including nausea [10], compared to men. In our study, both history of nausea related to migraine and other kind of headache were associated with the severity of NVP. Although headache, at least in a difficult form, may co-occur or cause nausea, it is possible that some women who answered having other type of headache had actually undiagnosed migraine. Theories behind higher prevalence of migraine in women include fluctuation in sex hormones, differences in sex hormone receptor binding and genetic factors [21, 29].

Previously, the use of contraception, especially oral combined contraception with higher hormonal doses, has been linked to nausea as a side effect [11, 22]. In a population-based study of 1000 women by Whitehead [25], women with NVP reported history of nausea related to the use of oral contraceptives. On the other hand, Gadsby et al. [26] did not find such connection in a smaller prospective study of 363 women. We found that women with history of nausea during the use of contraception also had higher odds for NVP. This finding, however, vanished in multivariable analysis. The low number of affected women, different formulas of contraception and lower hormonal doses in modern preparations could explain our neutral results.

There is increasing evidence about inheritance and genetics of NVP and HG [17], albeit genetics of NVP is less studied. In a study by Zhang et al. [13], women with HG were more likely to have mother and/or sisters with HG. Similar findings of high number of affected family members in women with HG were presented in a study by Fejzo et al. [15]. Moreover, the results of twin study by Colodro-Conde et al. [16] supported the increasing evidence of heritability of NVP. Our findings of association between NVP especially of first-degree relatives and the severity of women’s own NVP symptoms supported this evidence. Inheritance of HG is thought to be linked with maternal and possibly with fetal genotype and also placenta probably plays a role [6, 13, 17, 30, 31].

Our study has some limitations as well as some strengths. The data were collected retrospectively, when NVP symptoms had already relieved in most of the women. This could over- or underestimate the results. Nevertheless, we wanted to recruit women with large range of severity of NVP, also non-symptomatic women. Women start their follow-up in MHCCs typically at the end of the first trimester, when NVP usually has relieved. To test the effect of recall bias concerning the severity of NVP symptoms, we conducted a subgroup analysis of women with ≤ 20 gwk, who answered during NVP or right after the symptoms had relieved, with similar results. History of nausea variables were self-reported, and no medical records were available and thus the results should be interpreted with caution. With regards especially to migraine and motion sickness it is difficult to scale severity and labelling of the condition. On the other hand, having motion sickness is rarely reported in medical records. Concerning family history of NVP, questionnaire-based information may be prone to some degree of bias, since women with more severe NVP are probably more likely to inquire the experience of NVP in the relatives than women without NVP symptoms. This could also explain the low prevalence of affected second-degree relatives, given the population prevalence of NVP.

Our study is one of the few studies evaluating several aspects of history of nausea and risk of NVP. The merits of the study include high number of participants and the use of a structured questionnaire, the PUQE, previously validated to evaluate the severity of NVP [18]. Since the women in our study were enrolled from MHCCs which in Finland are free and attended practically by all pregnant women [19], they represent the general Finnish pregnant population.

All in all, NVP causes suffering to majority of pregnant women and especially severe NVP has clinical and economical importance. Today many pregnancies are carefully planned, and modern women seek individual counselling for their potential pregnancy complications. Accordingly, our findings give important information tools for health care personnel in pre-pregnancy patient guidance. Furthermore, these results can help women with history of nausea to prepare to cope with possible NVP and make personal arrangements needed for adapting to a possibly challenging early pregnancy beforehand.

## Electronic supplementary material

Below is the link to the electronic supplementary material.

Supplementary file1 (XLSX 28 kb)

Supplementary file2 (XLSX 28 kb)
